# Shoulder Rotator Muscle Dynamometry Characteristics: Side Asymmetry and Correlations with Ball-Throwing Speed in Adolescent Handball Players

**DOI:** 10.2478/hukin-2014-0059

**Published:** 2014-10-10

**Authors:** Inese Pontaga, Janis Zidens

**Affiliations:** 1 Department of Anatomy, Physiology, Biochemistry and Hygiene, Latvian Academy of Sports Education, Riga, Latvia.; 2 Department of Sport Games, Latvian Academy of Sports Education, Riga, Latvia.

**Keywords:** shoulder, internal and external rotator muscles, isokinetic dynamometry, handball, throwing speed

## Abstract

The aim of the investigation was to: 1) compare shoulder external/internal rotator muscles’ peak torques and average power values and their ratios in the dominant and non-dominant arm; 2) determine correlations between shoulder rotator muscles’ peak torques, average power and ball-throwing speed in handball players. Fourteen 14 to 15-year-old male athletes with injury-free shoulders participated in the study (body height: 176 ± 7 cm, body mass 63 ± 9 kg). The tests were carried out by an isokinetic dynamometer system in the shoulder internal and external rotation movements at angular velocities of 60°/s, 90°/s and 240°/s during concentric contractions. The eccentric external– concentric internal rotator muscle contractions were performed at the velocity of 90°/s. The player threw a ball at maximal speed keeping both feet on the floor. The speed was recorded with reflected light rays. Training in handball does not cause significant side asymmetry in shoulder external/internal rotator muscle peak torques or the average power ratio. Positive correlations between isokinetic characteristics of the shoulder internal and external rotator muscles and ball-throwing speed were determined. The power produced by internal rotator muscles during concentric contractions after eccentric contractions of external rotator muscles was significantly greater in the dominant than in the non-dominant arm. Thus, it may be concluded that the shoulder eccentric external/concentric internal rotator muscle power ratio is significantly greater than this ratio in the concentric contractions of these muscles.

## Introduction

One of the main aims of handball training is to increase ball-throwing speed. The shoulder joint internal rotation is one of the components of a throwing motion (Dillman et al., 1993; Van den Tillaar and Ettema, 2004; Werner et al., 2001). Sport specific training with regular, repeated throwing motions causes an increase of the shoulder internal rotator muscle torques in the dominant (throwing) arm, and the torques and power developed by these muscles are much higher than the shoulder external rotator muscle torques and power in the non-dominant arm (McMaster et al., 1991; Codine et al., 1997; Wilk et al., 1993).

One of the methods of measuring muscle torque and power is isokinetic dynamometry. The difference between the peak torque produced in the shoulder rotation motions by the dominant and non-dominant arms internal and external rotator muscles, respectively, in adult elite handball players evaluated by an isokinetic dynamometry is not significant at low (60°/s) or high angular velocity (180°/s, 240°/s) of the movements (Pontaga and Zidens, 2004). A significant difference is revealed between the dominant and non-dominant internal rotator muscles’ average power only at high angular velocity of the movements (240°/s). Therefore, asymmetry could appear also between the dynamometry characteristics of the dominant and non-dominant arm muscles in adolescent handball players.

To achieve high ball-throwing speed, a significant level of muscle strength and power are required. Maximal power, which is produced by arm muscles in a heavy weight horizontal bar press from a supine position does not differ significantly between handball players and weight lifters, while strength of the weight lifters is significantly greater (Izquierdo et al., 2002). A concentric shoulder adductor muscle’s peak torque value as measured by isokinetic dynamometry allows to predict ball-throwing speed for adult baseball players (Bartlett et al., 1989). Mikesky et al. (1995) could not find significant correlations between a baseball pitcher’s shoulder rotator muscle peak torques in the concentric and eccentric contractions measured by isokinetic dynamometry at the angular velocities of movement of 90°/s, 240°/s and 300°/s and ball throwing speed. Clements et al. (2001, a) compared the shoulder internal rotator muscle’s isometric strength and ball-throwing speed in adolescent elite baseball players and a control group of adolescent boys with the same height and body mass. The tests showed that high throwing speeds were achieved by well-trained athletes without changes of the isometric muscle strength in comparison with untrained adolescents. Clements et al. (2001, b) determined in adolescent baseball players a significant relationship between the isometric shoulder internal rotator muscle peak torque/body weight ratio and ball-throwing speed, and between isokinetic concentric elbow extensor muscle peak torque/body weight ratio at the angular velocity of 120°/s and throwing speed. Cohen and Mont (1994) determined a significant relationship between the tennis serve speed of elite tennis players and the dominant-arm shoulder internal/external rotator muscle peak torque ratio measured by an isokinetic dynamometer at the angular velocity of 60°/s and 180°/s in the concentric and eccentric contractions.

Many authors have investigated the shoulder internal–external rotator muscles because they are important to provide joint stability (Fleisig et al., 1995; Jobe and Pink, 1993). Nevertheless, shoulder external rotator muscle strength is important to provide stability of the shoulder joint during throwing motions, but a role of this muscle group in high ball-throwing speed in handball players has not been investigated.

The aims of our investigation were as follows: 1) to compare shoulder external/internal rotator muscle peak torques and average power values as well as their ratio in the dominant and non-dominant arm; and 2) to determine the correlations between shoulder rotator muscle peak torques, average power and ball-throwing speed in adolescent handball players.

## Material and Methods

Fourteen elite male adolescent handball athletes with seven years of training experience were informed of the aim and possible test risks of the study. The study was performed in accordance with the standards of the Ethics Committee of the Latvian Council of Sciences. All shoulder joints of the athletes were injury free and painless during the investigation. The average age, body height, body mass and body mass index of the tested athletes equaled respectively: 14.6 ± 0.8 years old, 176 ± 7 cm, 63 ± 9 kg, 20.3 ± 2.5 kg/m^2^.

The tests were carried out by the dynamometer system REV-9000 (Technogym, Gambettola, Italy). The shoulder internal rotation movement was performed by the pectoralis major, latissimus dorsi, teres major, deltoid (anterior fibers) and subscapularis muscles, but the external rotation movement was carried out by the infraspinatus, teres minor and deltoid muscles (Loudon et al., 1998). The athlete was seated on the bench with an elbow resting on an input shaft. A dynamometer level arm was adjusted to the length of the athlete forearm. The elbow was flexed to 90°. The humerus was abducted in a right angle (90°) to the trunk. The internal and external rotation movements were performed in the scapular plane, which lies parallel to the scapula plane surface. Greenfield et al. (1990) determined that testing was preferable in the scapular plane because the shoulder external rotational strength values in this plane were significantly higher than in the frontal plane.

Just before the investigation and between the tests of the muscles’ concentric–concentric and eccentric–concentric contractions, passive internal–external rotation motions in the shoulder joint were performed for 90 s at an angular velocity of 120°/s. The athletes were familiarized with the dynamometer while performing muscle contractions at submaximal force.

The range of movement (ROM) was from 20° of the shoulder external rotation to 100° of the internal rotation. To exclude trunk movement, the chest and pelvis were stabilized using straps. The handball player’s feet were placed on a support platform. The measurements were corrected for effects of gravity. The shoulder isokinetic internal and external rotation movements were tested at angular velocity values of 60°/s, 90°/s and 240°/s. Internal and external rotation movements were repeated five times at the velocity of 60°/s and 90°/s, respectively, and 20 times at the velocity of 240°/s. The passive internal–external rotation motions in the shoulder joint were performed for 90 s at the angular velocity of 120°/s between the tests of different velocities.

The muscle contractions were concentric at all rotation velocity values, but at the velocity of 90°/s, additional eccentric external rotator– concentric internal rotator muscle contractions were performed. Peak torque values (N.m) of the internal and external rotator muscles were obtained from the best repetition (greatest peak torque). The average power (W) of the internal and external rotator muscles was determined from all movements of each angular velocity.

Before the throwing test, the athletes performed a general warm-up for 15 minutes. The warm-up included seven minutes of low- to moderate-intensity jogging, jumping rope and light shoulder and arm exercises; four minutes of general active stretching exercises for the main muscle groups; four minutes of specific active stretching exercises for shoulder, arm and hand muscles.

Ball-throwing speed (m/s) was measured by a Superschus (EDV-Beratung Arbeiter, Bremen, Germany). The handball player threw a 0.4 kg ball using the dominant arm at maximal speed six times with both feet on the floor. The distance between the athlete and the Superschus was 2.5 m. The speed was recorded with reflected light rays. The Superschus consisted of two stands. Every stand contained two vertically oriented light ray sources (generating six light rays) or sensors (detecting the six light rays), respectively. The distance between the measuring sensors was 0.8 m. The thrown ball was detected at the height of flight from 1.7 m to 2.5 m from the floor. The ball throwing speed was calculated automatically by the device at this 0.8 m flying distance between the sensors. The best result (highest speed of the ball) was used for further analysis.

Mean values and standard deviations for all characteristics were calculated. A dependent t-test for paired data groups was employed to determine differences between the peak torques and average power levels produced by the shoulder rotator muscles of the dominant and non-dominant arms. The differences were considered statistically significant at p < 0.05. A correlation analysis was performed to determine the relationships between the dynamometry characteristics of the dominant arm’s shoulder rotator muscles and the ball-throwing speed. Microsoft Excel 2007 was used to perform all statistical procedures.

## Results

**Figure 1:**
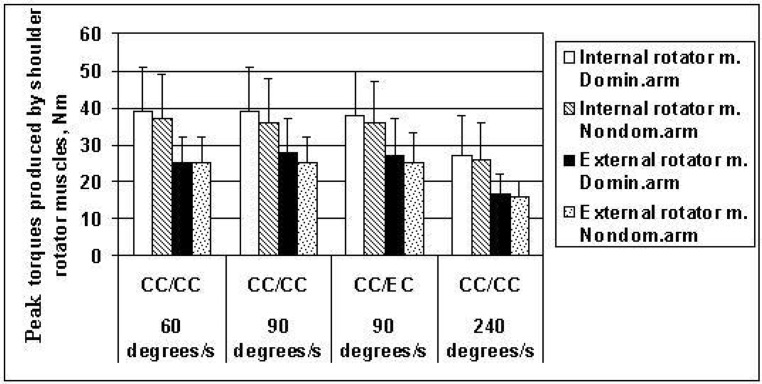
Peak torques (N·m) developed by shoulder internal and external rotator muscles at angular velocities of movement at 60°/s, 90°/s and 240°/s in the concentric contractions (CC/CC) and concentric internal/eccentric external (CC/EC) rotator muscle contractions

The differences between the peak torques of the shoulder internal and external rotator muscles of the dominant and non-dominant arms were not statistically significant at 60°/s, 90°/s or 240°/s in the concentric contractions of muscles, nor in the eccentric contractions of the external rotator muscles at 90°/s (Figure 1). This means that asymmetry of the shoulder rotator muscles strength in the dominant and non-dominant arms was not significant in adolescent handball players.

**Figure 2:**
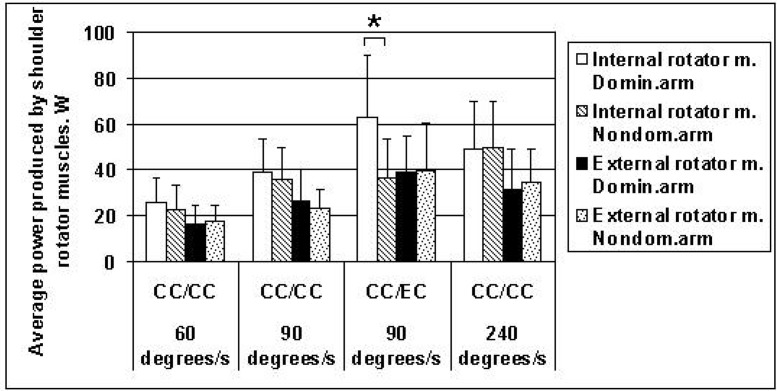
Average power (W) produced by shoulder internal and external rotator muscles at angular velocities of movement of 60°/s, 90°/s and 240°/s in the concentric contractions (CC/CC) and concentric internal/eccentric external (CC/EC) rotator muscle contractions

The average power produced by the shoulder internal rotator muscles in the dominant and non-dominant arm did not differ significantly at different velocities of the movement (60°/s, 90°/s and 240°/s) in the concentric contractions of both groups of the shoulder rotator muscles (Figure 2). The average power produced by the shoulder internal rotator muscles in the concentric contractions after eccentric contractions of the external rotator muscles at 90°/s was significantly greater in the dominant arm in comparison with the non-dominant arm (Figure 2). The reason for this asymmetry was better adaptation of the dominant arm’s shoulder rotator muscles to the regular throwing motion. The difference between the shoulder external rotator muscle-produced average power of the dominant and non-dominant arms was not statistically significant at any velocity of movement in the concentric contractions of muscles or at 90°/s in the eccentric contractions of the muscles (Figure 2).

The ratio of shoulder external/internal rotator muscle peak torque values did not differ significantly between the dominant and non-dominant arms, and did not depend on the velocity of rotation movement (Table 1). In the dominant arm, this ratio varied from 0.66 to 0.70, but in the non-dominant arm it varied from 0.64 to 0.72. The external/internal rotator muscle-produced average power ratio values were also similar in the dominant and non-dominant arms (Table 1). This ratio in the dominant arm varied from 0.68 to 0.74 and in the non-dominant arm from 0.69 to 0.71 in the concentric contractions of shoulder rotator muscles and again did not depend on the velocity of movement. The shoulder external/internal rotator muscles’ average power ratio was 0.94 in the eccentric contractions of the external rotator muscles and concentric contractions of the internal rotator muscles. This ratio was significantly greater than in concentric contractions of both groups of shoulder muscles. The shoulder external rotator muscles’ eccentric action is especially important in joint stability and for generating higher ball speed in handball players.

Average maximal ball-throwing speed in adolescent handball players was 21.01 ± 1.37 (m/s). The highest peak shoulder internal and external rotator muscles' torques were observed at the slow (60°/s) and medium (90°/s) rotation speeds (Table 2 and Figure 1). Average power values produced by the shoulder internal and external rotator muscles were greatest in the concentric contractions of internal rotators after eccentric action of the external rotator muscles at 90°/s and 240°/s (Table 2 and Figure 2).

Positive correlations between the isokinetic characteristics of the shoulder rotator muscles and the ball-throwing speed were observed (Table 2). The values of correlation coefficients between the peak torques, average power values of shoulder internal and external rotator muscles and the ball-throwing speed were similar for both muscle groups at 60°/s and 90°/s. The correlation between the shoulder external rotator muscles peak torques and the ball-throwing speed was slightly higher (r = 0.69) than the correlation between the shoulder internal rotator muscle peak torques and the ball-throwing speed (r = 0.61) at 240°/s. The correlations between average power produced by shoulder rotator muscles and ball-throwing speed were similar for shoulder internal and external rotator muscles at 240°/s.

## Discussion

Regular, repeated throwing motion in many sports causes selective development of the shoulder muscles in the dominant arm. Brown et al. (1988) investigated the shoulder rotation by an isokinetic device in professional baseball players. They detected differences in the range of shoulder motions in the dominant and non-dominant arms (for example, more shoulder external rotation in the dominant arm). They observed a difference between the pitchers and position players. Shoulder internal and external rotator muscles of the dominant arm produced greater torque in comparison with the muscles of the non-dominant arm at fast isokinetic velocities of movement (180°/s, 240°/s and 300°/s). We did not observe shoulder external/internal rotator muscle peak torque ratio asymmetry between arms. The peak torques produced by the shoulder internal rotator muscles were significantly greater in the dominant arm than in the non-dominant arm muscles in elite water polo players at 30°/s (McMaster et al., 1991). The external rotator muscles had not increased peak torque values, while internal rotators were overdeveloped due to repetitive swimming and throwing motions. This can explain the shoulder muscles’ strength balance alteration and the development of instability of the joint. The external/internal rotator muscles’ peak torques ratio in water polo players (from 0.61 at the angular velocity of 30°/s to 0.55 at the velocity of 180°/s) was lower than in the control group (from 0.78 at the angular velocity of 30°/s to 0.65 at the velocity of 180°/s), but differences between the sides were not observed. Codine et al. (1997) investigated shoulder internal–external rotation using an isokinetic device in nonathletes, runners, tennis players and baseball players at the velocities of 60°/s, 180°/s and 300°/s. The shoulder rotator muscles’ peak torque ratios in the untrained group and runners were similar. The tennis players had greater torques produced by internal rotator muscles, but baseball players showed the most altered rotator muscle peak torque ratios. Controversially, no significant differences in concentric strength were observed between the dominant and non-dominant arms for shoulder internal and external rotation in baseball pitchers (Mikesky et al., 1995; Bartlett et al., 1989; Wilk et al., 1993) and adult handball players (Pontaga and Zidens, 2004). Mikesky et al. (1995) offered several explanations: differences in testing positions, testing velocity, range of motion, sport specialization and sport level. The present results are in agreement with the data of these authors (Mikesky et al., 1995; Bartlett et al., 1989; Wilk et al., 1993) and the data obtained in adult handball players (Pontaga and Zidens, 2004). In our investigation of adolescent handball players in the concentric contractions of the shoulder internal and external rotator muscles, no significant asymmetry between the dynamometry characteristics of the internal rotator muscles of the dominant and non-dominant arms was detected. The lack of isokinetic characteristics asymmetry can be explained by the large difference between the isokinetic shoulder motions in the tests and the real throwing movement. The isokinetic motions are slower than the throwing movement. Even the fastest isokinetic velocities (450–500°/s) do not approach the joint angular velocity recorded in actual throwing motions (Malone and Garret, 1996). Dillman et al. (1993) and Werner et al. (2001) have determined the relationships between an arm throwing kinematics and the shoulder motions of baseball pitchers using three-dimensional, high-speed video data. Werner et al. (2001) detected a shoulder internal rotation mean velocity of 8286°/s, but according to Dillman et al. (1993) this value was 6940°/s. These velocities are much higher than the highest angular velocity used in our study (240°/s). We did not apply the slow angular velocity because it was not easy for the adolescent handball players to tolerate a high level of the muscles exertion required in the slow (30°/s) isokinetic exercises. The risk of injury is great also in the eccentric contractions at high velocities (Cohen et al., 1994), therefore, we used only medium angular velocity (90°/s) in the eccentric contractions of muscles.

In our present investigation, the average maximal ball-throwing speed in adolescent handball players was 21.01 ± 1.37 (m/s). The maximal ball throwing speed in adult handball players is greater - 26.4 ± 1.5 (m/s) in amateur players (Pontaga and Zidens, 2012) and 23.8 ± 1.9 (m/s) in professional Spanish players (Gorostiaga et al., 2005). The difference can be explained by greater strength of the muscles participating in the throwing motion and longer training experience in adult athletes in comparison with the adolescents.

A significant difference between the average power produced by the internal rotator muscles measured by the isokinetic dynamometry of the dominant and non-dominant arms was determined only in the concentric contractions of the internal rotator muscles after the eccentric contractions of the external rotator muscles (p < 0.05). Jobe et al. (1983) obtained similar shoulder muscle asymmetry in concentric–eccentric muscle contractions in male amateur baseball players. They detected an insufficiency of the shoulder rotator muscles’ (deltoid, subscapularis, infraspinatus and teres minor muscles) activity in dynamic electromyography during the acceleration stage of the throwing and pitching motions. It was observed only in the eccentric contractions of the muscles. Throwing is a complex task that requires coordinated motions of the legs, trunk and arms. The shoulder external rotation range produced by the isokinetic dynamometers is small in comparison with a real throwing movement, where elite athletes reach up to 170° of shoulder external rotation (Dillman et al., 1993). In our investigation, the average power of shoulder internal rotator muscles was significantly greater in the concentric contractions after the eccentric contractions of the external rotator muscles than in solely concentric contractions only for the dominant arm. This can be explained by eccentric shoulder muscles action role in performance determination of different types of throws in handball to maintain the shoulder joint stability in the range of movements. The deceleration stage of throwing represents the most violent phase of the throwing motion. Deceleration occurs from the point of ball release to the point of 0° of rotation. It is a point of marked eccentric contraction of the rotator cuff to slow down the arm motion and is a point of maximal shoulder joint posterior capsule stress. Joint loads approach posterior shear of 400 N, inferior shear of 300 N, and compressive forces of nearly 1,000 N (Jobe et al., 2000). Stickley et al. (2008) examined the shoulder external/internal rotator muscles’ peak torque ratio in the dominant arm of adolescent female volleyball athletes. They determined that previously injured athletes produced significantly lower eccentric external rotation to concentric internal rotation strength ratios compared with volleyball players without an injury history (p < 0.04). Therefore, these authors recommended preventive shoulder strengthening programs focused on improving eccentric strength and correcting imbalances between internal and external rotator muscles for all female adolescent volleyball athletes.

From the above data, it seems that the main muscles providing the shoulder joint stability during throwing movement are the rotator cuff muscles (internal and external rotators). This supports the findings of Latimer et al. (1998), who investigated activation time and pattern of shoulder muscles by electromyography after application of an anterior translation force on the shoulder joint. Anterior shoulder muscles (anterior part of deltoid muscle, pectoralis major muscle and long head of biceps muscle) activated before the posterior muscles (latissimus dorsi muscle), and the rotator cuff muscles fired with greater magnitude than the other groups of muscles. This confirms an important role of rotator cuff muscles in joint stability provided during shoulder movements.

Therefore, throwing athletes must strengthen the rotator cuff muscles in the rehabilitation process after shoulder injuries, as must non-injured athletes to prevent the shoulder instability. Beneka et al. (2002) investigated the effect of shoulder muscle training using exercises over a period of six weeks: one group of young healthy people trained following an isokinetic exercises program, another group followed multi-joint dynamic resistance training for the rotator cuff muscles. Statistically significant improvement in the internal and external rotator muscles strength was observed after both training programs. Therefore, six weeks of training of shoulder rotator muscles improved their strength, and isokinetic dynamometry was a useful tool for controlling this training process.

Changes in the shoulder rotator muscle torque ratio may cause dislocation of shoulder articulating surfaces. The most common complication of this is recurrence, which occurs much more often in adolescent athletes (Walton et al., 2002). In the present study, shoulder external/internal rotator muscle peak toque values in the adolescent handball players (from 0.64 to 0.72 for both arms) were lower than these ratios values in adult handball players (close to 0.80) (Pontaga and Zidens, 2004). This confirms that shoulder external rotator muscle strengthening exercises must be included in the training program of adolescent handball players.

Our investigation suggests that high shoulder internal and external rotator muscle strength and power values are important to reach high ball-throwing speed in handball. This means that training of shoulder external rotator muscles is important not only to improve shoulder joint stability during throwing motion but also to increase the ball-throwing speed in adolescent handball players.

## Conclusions

1. Training in handball did not cause significant asymmetry in shoulder external/internal rotator muscles peak torques and average power values between the dominant and non-dominant arms at different velocities of movement: slow (60°/s) medium (90°/s) and fast (240°/s) in the concentric contractions of muscles and at 90°/s in the eccentric contractions of the external rotator muscles.
2. The ratio of shoulder external/internal rotator muscle peak torque values did not differ significantly between the dominant and non-dominant arm, and did not depend on the velocity of rotation movement (this ratio varied from 0.64 to 0.72). The external/internal rotator muscle-produced average power ratio values were also similar between the dominant and non-dominant arms (this ratio varied from 0.68 to 0.74) in the concentric contractions of shoulder rotator muscles and did not depend on the velocity of movement.
3. The average power produced by the shoulder internal rotator muscles in the concentric contractions after eccentric contractions of the external rotator muscles at 90°/s was significantly greater in the dominant arm in comparison with the non-dominant arm. The shoulder external/internal rotator muscle average power ratio was 0.94 in the eccentric contractions of the external rotator muscles and concentric contractions of the internal rotator muscles. This confirms that the shoulder external rotator muscle eccentric action is especially important in the joint stability and ball-throwing speed in handball players.
4. We detected positive correlations between the isokinetic characteristics of the shoulder rotator muscles and the ball-throwing speed. This indicates that the contribution to the shoulder internal and external rotator muscles peak torques and average power production is at least similar in high ball-throwing speed achievement. The correlation between theshoulder external rotator muscle peak torques and the ball-throwing speed was slightly closer (r = 0.69) than the correlation between the shoulder internal rotator muscle peak torques and the ball-throwing speed (r = 0.61) at the fast velocity of movement (240°/s).

## Figures and Tables

**Table 1:** The shoulder external/internal rotator muscle peak torques and average power ratios at different isokinetic angular velocities of movement and muscle contractions (concentric contractions of both rotator muscle groups (CC/CC) and eccentric external/concentric internal rotator muscle (EC/CC) contractions) in adolescent handball players

Velocity of movement	Muscle contractions	Peak Dominant arm	torque Non-dom. arm	ratio Diff., signif.	Average Dominant arm	power Non-dom. arm	ratio Diff., signif.
60°/s	CC/CC	0.66± 0.11	0.69± 0.13	5 %	0.68± 0.16	0.71± 0.15	4 %
				N.S.			N.S.
90°/s	CC/CC	0.70± 0.09	0.72± 0.15	3 %	0.72^*^ ± 0.15	0.69^*^ ± 0.14	4 %
				N.S.			N.S.
90°/s	EC/CC	0.69± 0.12	0.69± 0.13	0 %	0.94^*^ ± 0.26	0.94^*^ ± 0.25	0 %
				N.S			N.S.
240°/s	CC/CC	0.67± 0.14	0.64± 0.15	4 %	0.74± 0.10	0.70± 0.11	5 %
				N.S.			N.S.

* Significant differences between the shoulder’s external/internal rotator muscle average power ratios in the CC/CC and EC/CC contractions in the dominant and non-dominant arm, respectively, at the velocity of movement of 90°/s (p ≤ 0.004)

N.S. – not significant

**Table 2:** The dominant arm’s shoulder internal rotator (IR) muscle and external rotator (ER) muscle peak torques (τ_peak_) and average power (P) at different angular velocities of shoulder internal–external rotation movements in concentric muscle contractions and eccentric (EC) ER–concentric (CC) IR contractions, and the correlation coefficients (r) between these isokinetic characteristics and ball-throwing speed

Velocity	60°/s, concentric contractions		90°/s, concentric contractions		90°/s, EC ER, CC IR c.		240°/s, concentric contractions	

Characteristic								
τ_peak_ IR (Nm)	40±11	r=0.85^*^	39± 10	r=0.76^*^	38 ± 10	r=0.77^*^	27 ± 11	r=0.61^*^
P. IR (W)	27 ± 8	r= 0.77^*^	37± 12	r=0.78^*^	51 ± 19	r=0.62^*^	48 ± 20	r=0.66^*^
τ_peak_ ER (Nm)	26 ± 6	r= 0.83^*^	29 ± 9	r=0.75^*^	28 ± 9	r=0.76^*^	17 ± 5	r=0.69^*^
P, ER (W)	18 ± 5	r= 0.74^*^	28 ± 9	r=0.77^*^	38 ± 15	r=0.79^*^	33 ± 13	r=0.67^*^

* - probability level p ≤ 0.02
